# A Process Evaluation Examining the Performance, Adherence, and Acceptability of a Physical Activity and Diet Artificial Intelligence Virtual Health Assistant

**DOI:** 10.3390/ijerph17239137

**Published:** 2020-12-07

**Authors:** Courtney R. Davis, Karen J. Murphy, Rachel G. Curtis, Carol A. Maher

**Affiliations:** 1 City East Campus, Alliance for Research in Exercise, Nutrition and Activity, University of South Australia, GPO Box 247, Adelaide 5001, Australia; courtney.davis@unisa.edu.au (C.R.D.); karen.murphy@unisa.edu.au (K.J.M.); rachel.curtis@unisa.edu.au (R.G.C.); 2 City East Campus, University of South Australia, Clinical and Health Sciences, GPO Box 247, Adelaide 5001, Australia; 3 City East Campus, University of South Australia, Allied Health and Human Performance, GPO Box 247, Adelaide 5001, Australia

**Keywords:** virtual health assistant, conversational agent, chatbot, lifestyle, intervention, Mediterranean diet, physical activity, process evaluation

## Abstract

Artificial intelligence virtual health assistants are a promising emerging technology. This study is a process evaluation of a 12-week pilot physical activity and diet program delivered by virtual assistant “Paola”. This single-arm repeated measures study (*n* = 28, aged 45–75 years) was evaluated on technical performance (accuracy of conversational exchanges), engagement (number of weekly check-ins completed), adherence (percentage of step goal and recommended food servings), and user feedback. Paola correctly asked scripted questions and responded to participants during the check-ins 97% and 96% of the time, respectively, but correctly responded to spontaneous exchanges only 21% of the time. Participants completed 63% of weekly check-ins and conducted a total of 3648 exchanges. Mean dietary adherence was 91% and was lowest for discretionary foods, grains, red meat, and vegetables. Participants met their step goal 59% of the time. Participants enjoyed the program and found Paola useful during check-ins but not for spontaneous exchanges. More in-depth knowledge, personalized advice and spontaneity were identified as important improvements. Virtual health assistants should ensure an adequate knowledge base and ability to recognize intents and entities, include personality and spontaneity, and provide ongoing technical troubleshooting of the virtual assistant to ensure the assistant remains effective.

## 1. Introduction

There is a global rise in disease burden from cardiovascular disease and diabetes—chronic conditions which are predominantly modifiable by healthy diet and lifestyle choices [1]. In Western nations, poor diet quality and suboptimal physical activity levels contribute significantly to the burden of disease [2]. In Australia, adults consume a third of their energy from discretionary foods, less than 1 in 20 adults consume the recommended amount of fruits and vegetables [3], and two-thirds of the adult population are overweight or obese [4].

Preventative measures to address these behavioral risk factors could reduce the burden of disease significantly [5]. However, interventional strategies to improve diet quality and increase physical activity are difficult for individuals to undertake alone without the support of a health professional. Consulting with a health professional currently involves one-on-one repeat visits which can be costly to both the health system and the individual [6]. Artificially intelligent virtual assistants are ubiquitous in society today. Prominent examples are Amazon’s Alexa or Apple’s Siri [7], where users can access these virtual assistants 24-h a day and receive personalized and instant support. The appeal of this type of intervention in a health context is clear. Virtual assistants, along with other e- and mHealth technologies, such as websites, videos, and fitness trackers, provide an opportunity for cost-effective, scalable, and wide-reaching interventions to address sub-optimal lifestyles [6,8].

The field of research into virtual health assistants is expanding rapidly. In 2020 alone, virtual assistants or chatbots have been tested for pain-self management amongst adults with chronic pain [9], promoting fertility awareness among child-bearing women [10], for life skill promotion amongst adolesents [11], and to promote physical activity amongst office workers [12]. Other recently tested virtual assistants include Tess to support adolescents with pre-diabetes [7] and Woebot to support adolescents with depression and anxiety [13]. There are fewer examples of vitual assistants with a focus on physical activity and diet. Bickmore et al. [14] tested a virtual assistant for improving physical activity and fruit and vegetable intake, as well as physical activity in older adults [15]. Reflection Companian was used to promote physical activity amongst adults based on FitBit data [16]. All of these studies were pilot in nature, often of short duration (2–12 weeks) and small sample size. While all showed at least some improved outcomes for those exposed to the virtual assistant, most had limited process evaluation, if any.

These studies have been collated in three recent reviews [7,17,18]. In 2018, a systematic literature review identified 14 studies involving artificial intelligence (AI) virtual health assistants capable of natural language processing [7]. Overall, the heterogeneity of the virtual assistants, study design, and evaluation tools provided inconclusive evidence of the potential benefit of virtual assistants for changing behavior. Although some individual studies showed positive user satisfaction and some positive changes in health-related measures, none of the studies in the systematic review focused on physical activity or dietary behavior. A review published in 2019 focused on the use of virtual assistants in treatment of mental health, including 13 studies [17]. The authors of this review also noted good success of the virtual assistant, particularly versus a non-active control group, but high heterogeneity between studies in terms of design and outcomes. Finally, a review completed in July 2020 focused specifically on virtual assistants designed to improve diet and physical activity, which included the present study and 6 others [18]. All results suggested efficacy of using a virtual health assistant for promotion of healthy diet and physical activity. All reviews noted a lack of consistent reporting on feasibility and user evaluation of the virtual assistant.

Being such a fledgling field of research, to our knowledge, no natural language processing virtual assistants designed to assist users to undertake lifestyle changes (such as physical activity or diet) have undergone thorough process evaluation, examining aspects such as the technical performance of the virtual health assistant, user engagement and adherence, and user experience over time. Such information is essential to understand the potential utility of this type of technology for delivering effective health behavior interventions and to identify critical areas for improvement. Given that this is a limitation highlighted by several recent systematic reviews in this field, this study set out to examine engagement with, and performance of a virtual health assistant, named “Paola”, created to deliver a lifestyle intervention for older adults.

Paola was recently evaluated in a 12-week single-arm, pre-post pilot study [19]. The present study aimed to examine:

1. The technical performance of the virtual health assistant;
2. Participant adherence with weekly physical activity and dietary goals;
3. User feedback on the virtual health assistant;
4. The association between engagement with the virtual health assistant and primary trial outcomes (12-week change in physical activity and diet score).

## 2. Materials and Methods

The study was approved by the University of South Australia Human Research Ethics Committee (#201724) and prospectively registered with the ANZCTR (#ACTRN12619000015145). All participants provided informed consent before commencing the study.

The current study was a secondary analysis process evaluation of a 12-week lifestyle physical activity and healthy eating (based upon the Mediterranean dietary pattern) program delivered via a virtual health assistant in a single-arm repeated measures study. Full details of the virtual health assistant, study methods, and primary outcomes have been published elsewhere [19]. Briefly, over the 12-week study period, participants significantly increased their self-reported weekly moderate-to-vigorous physical activity (MVPA) by 110 min, increased their diet quality scores by 150%, lost 1.3 kg in body weight, and lost 2.1 cm from their waist circumference (all *p* < 0.05) [19].

### 2.1. Intervention Description

The virtual health assistant (“Paola”) delivered personalized physical activity and dietary coaching through:

1. An induction session which taught users about increasing physical activity and the Mediterranean diet (characterized by plentiful vegetables, fruits and whole grains, healthy proteins (e.g., fish, seafood, and legumes), healthy fats (e.g., nuts, olive oil) and herbs and spices instead of added salt), including recommended daily and weekly servings goal-setting and self-monitoring [20];
2. Every week after the induction session, for 11 weeks, participants received an email prompting them to complete a weekly check-in with Paola regarding their physical activity and diet for the past week. Paola provided personalized feedback, and then assisted users in setting personalized step goals for the coming week;
3. Paola was available 24/7 to answer users’ questions regarding physical activity and the Mediterranean diet.

The virtual assistant Paola was built using International Business Machines (IBM’s) Watson Virtual Assistant AI software. Participants interacted with Paola using natural language through a text-based conversation. Paola uses a mixture of dialogue management strategies, including finite-state (baseline initiation session), frame-based (weekly check-in), and agent-based (answering questions).

Paola referred to users by their first name and could respond to questions at any time. Working in collaboration with the private artificial intelligence software specialists who were engaged to create the software, Paola was designed to respond to users’ spontaneous questions based on the questions’ “intent” and “entity”. Intents and entities were derived from a list of frequently asked questions experienced by dietitians and physiotherapists in previous diet and exercise trials. Commonly raised topics, such as olive oil, formed the entities, while sub-entities included more specific topics such as cooking with olive oil. Intents were categorized into types, such as “how much” type questions, and multiple utterances for each intent were programmed into Paola’s logic; for example, she would recognize “what amount” and “how much” as the same intent. Paola had the ability to recognize pairings of intents and entities, with a further deviation for sub-entities, with a different programmed response option for most combinations. The response options were written specifically for each pairing and programmed into the software.

**Figure 1:**
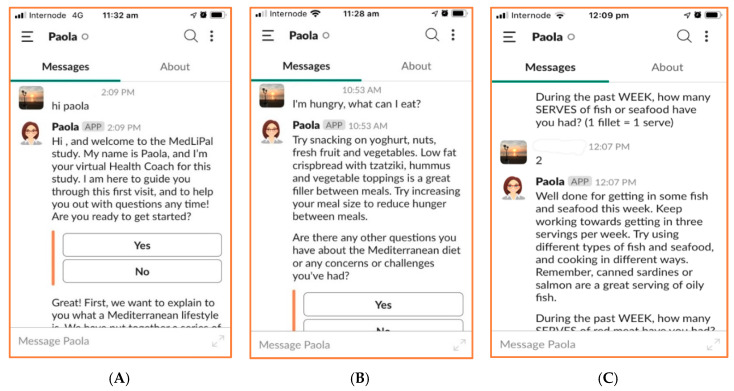
Screenshots of interactions with Paola via Slack application. Panel (**A**): The beginning of the baseline initiation. Panel (**B**): Paola responding to a question. Panel (**C**): Weekly check-in process.

Paola was deployed on the cloud-based instant messaging platform Slack (Slack Technologies; Figure 1). She was designed to be used in concert with a wrist-worn activity tracker (Garmin Vívofit 4) and purpose-designed website, which housed educational videos which Paola directed users to watch during the induction session, as well as several recipes. Participants also received a weekly log-sheet to log daily step count and dietary intake.

### 2.2. Participants and Procedure

Participants were middle-aged and older adults, aged 45–75 years, who owned a smart-phone, and who did not currently follow a Mediterranean dietary pattern or meet guidelines for physical activity (minimum 150 min of moderate activity per week). They were recruited through mainstream media news stories, flyers, and free social media posts.

Participants attended three face-to-face appointments at the University of South Australia Clinical Trials Unit (at 0, 6, and 12 weeks). In the baseline visit, participants completed the induction session with Paola and were provided with all study equipment (wrist-worn wearable and weekly log sheets). In all three appointments, they completed the main study outcome measures (self-reported physical activity, self-reported Mediterranean diet quality, and had their weight, waist circumference, and blood pressure measured objectively), and a feedback questionnaire gathering their experiences and perspectives about using Paola.

### 2.3. Outcomes

The total minutes of weekly MVPA was assessed using the Active Australia Survey (AAS), a widely-used, validated, eight-item questionnaire which collects frequency and duration of vigorous physical activity, walking, and other moderate physical activity [21,22]. Mediterranean diet adherence was measured using a 14-item Mediterranean diet adherence tool, adapted from the Prevención con Dieta Mediterránea (PREDIMED) study [23] to align with the Australian food supply (Australian version validated relative to Mediterranean diet score calculated from 3-day weighed food record *r* = 0.44) [24]. Dietary adherence is expressed as a score out of 14, with higher scores indicating higher adherence.

Each participant created a Slack user account, which assigned them a unique identifying number. All occasions of engagement with Paola were then automatically recorded, including:

- The type of interaction with Paola (i.e., the induction session, weekly check-ins, and spontaneous exchanges);
- Data entered by participants (e.g., their weekly physical activity (step count) goal, weekly dietary and step data entered during the weekly check-in, and questions (verbatim) they asked of Paola);
- Paola’s full dialogue (verbatim);
- The back-end data regarding what Paola recognized as entities and intents in participants’ spontaneous questions.

All interactions were time and date stamped. After the study concluded, the server logs were downloaded into Microsoft Excel spreadsheets.

The virtual health assistant’s technical performance was scrutinized by examining server logs to determine whether she responded appropriately to participants during the weekly check-ins and spontaneous exchanges. A single weekly check-in involved a total of 34 dialogue exchanges between Paola and the participant (e.g., where Paola asked the participant about their step count and dietary intakes for the previous week, the participant entered their data, and Paola provided tailored feedback). Two indicators of technical performance were calculated: (1) Per cent accurate exchanges during weekly check-ins, and (2) per cent accurate responses to spontaneous exchanges.

Engagement data were collapsed to create two engagement metrics: (1) The number of weekly check-ins completed, and (2) the number of exchanges with Paola.

Adherence was determined based on weekly physical activity and dietary data that participants reported to Paola during the weekly check-in sessions. Participants were asked to set themselves a daily step goal, which provided a target for the week. Each week this goal was reviewed and could be raised or lowered. For each participant, we recorded weekly step count goal and actual weekly steps taken, and weekly intakes of the Mediterranean dietary components. We then calculated the following adherence metrics: (1) Percentage of weekly physical activity goal achieved, and (2) percentage of recommended dietary intake achieved, by comparing actual servings with recommended servings.

User feedback was captured in online feedback surveys that participants completed at the end of the induction session, and again at 6 and 12 weeks. The surveys sought participants’ feedback on how useful they found the various program components, likes, dislikes, and suggestions for improvement.

### 2.4. Statistical Analysis

Most analyses were descriptive in nature, for example, percentages of actual vs. target physical activity and dietary intake behaviors, and percentage of participants’ feedback offering agreement with statements regarding the program’s usefulness.

For the accuracy of spontaneous exchanges with Paola, exchanges were grouped by theme (diet, physical activity, the program, other). We identified the percentage of Paola’s responses that were appropriate and examined how intents and entities were recognized by Paola to determine why Paola provided incorrect responses.

Engagement with Paola and program adherence (step count and dietary intakes) across the 12-week program duration were presented graphically. Visual inspection was then used to identify trends for change in engagement and adherence over time.

An exploratory analysis was undertaken to examine associations between engagement with Paola (based on the number of weekly check-ins completed) and overall study outcomes (change in physical activity and diet quality from baseline to 12 weeks). For these analyses, we categorized participants as high or low engagers based on median splits (high engagers completed ≥8 check-ins, low engagers completed <8 check-ins). Two linear mixed-effects models were constructed; the first with MVPA as the depending variable, and the second with the dietary adherence score as the dependent variable. These analyses were conducted using Stata version 15, with a *p*-value of 0.05 used to denote statistical significance.

## 3. Results

Participants were 31 adults aged 45–73 years (mean age 56.2 ± 8; 68% female). Of the 31 participants who commenced the program, 29 remained enrolled at week 6, and 28 completed the program (week 12).

### 3.1. Aim 1: Technical Performance

#### 3.1.1. Scripted Conversations

Males and females completed a similar number of check-ins (mean of 6.8/11 for females and mean of 7/11 for males). During each weekly check-in, Paola asked a series of questions to determine the participant’s recent diet and physical activity. Analysis of the weekly check-ins indicated that Paola asked the required questions correctly 97% of the time. Paola also provided feedback to the participant based on their responses to these questions (e.g., if the participant reported consuming two tablespoons of olive oil per day, Paola would respond with “Well done, you have had 2 servings of olive oil per day. You are on track following the Mediterranean diet!”). Analysis of the weekly check-ins indicated that Paola provided feedback correctly 96% of the time.

Errors during check-ins appeared to be caused by time-out issues. Slack considers a user inactive after 10 min of no curser activity [25]. This caused Paola to either restart the check-in or to freeze and provide no response after 10 min of inactivity.

#### 3.1.2. Spontaneous Exchanges

Spontaneous exchanges occurred when participants asked a question of the virtual health assistant or made a statement that was not part of the pre-defined script of the weekly check-in. This included interactions occurring outside the weekly check-in, initiating the check-in, and questions asked during the check-in (but not responses to Paola’s questions about diet or physical activity during the check-in). Due to a technical error, we were able to access transcripts for only 45 exchanges for analysis, though discussion with participants suggested that more exchanges occurred. Supplementary File 1 (Tables S1–S4) shows the participant input (question or statement) and Paola’s response, grouped into four tables (1) diet, (2) physical activity, (3) program, and (4) other.

There were 20 exchanges related to diet (Table S1). Paola provided an appropriate response for four exchanges (20%), though it is difficult to know if responses to “green vegetables?” (#3) and “pumpkin?” (#4) truly answered the participants query, as these queries were non-specific.

In some instances, server logs indicated that Paola recognized a relevant entity (e.g., #15 fruit, #16 fish) but misunderstood the intent of the question (identified intent #15 is consumption allowed, #16 how much). She therefore provided a response that was related to the entity but did not actually answer the question. When this occurred on some occasions during the weekly check-in, Paola simply continued with the next programmed statement instead of providing a response (e.g., #13–14). Due to the limitations of the programming, Paola was unable to recognize any entity or intent in some exchanges and provided no response or stated she could not answer (e.g., #7–9).

Participants demonstrated some flexibility and understanding of conversational programming when they attempted to ask the same question in similar ways (e.g., #7–9 were consecutive exchanges by one user, as were #10–11 and #13–14); however, they were ultimately unsuccessful as Paola was not programmed to respond to these particular queries, regardless of how they were asked. Only one question was asked related to physical activity (Table S2), which Paola was able to answer correctly.

There were 21 exchanges relating to the program (Table S3), 13 of which were to initiate the weekly check-in. Seven attempts (54%) to initiate the weekly check-in were successful. Five attempts to initiate the check-in were incorrectly recognized as asking for advice about the check-in. While Paola recognized the correct intent from statements such as “can I”, “may I”, “ready”, and “start”, she did not appear to understand intents such as “I want to” or “I would like to”. Paola also did not recognize “sign in” as referring to the weekly check-in and provided no response.

Six exchanges were made to correct information given to Paola in the weekly check-in, or to complain about the weekly check-in. Three of these statements contained the word “check-in” and were incorrectly recognized as asking for advice about the check-in (#35–37). Two exchanges attempted to correct information immediately after the participant’s mistake, but Paola could not recognize this and continued with the next statement programmed in the weekly check-in (#38–40).

Participants made three statements that could not be categorized under diet, physical activity or the program (#43–45, shown in Table S4). These statements were reasons provided during the weekly check-in as to why the participants had not met their goals. Paola was programmed to answer questions related to diet, physical activity, and the program, but was not programmed to provide understanding or empathy. She did not recognize an entity or intent for these items and was therefore unable to respond.

Although 35% of the sample were male, only 13% of spontaneous interactions were initiated by males. Of the 6 spontaneous interactions by males, 3 were diet questions, 2 were program related, and one was related to other themes (Supplementary Table S1).

### 3.2. Aim 2: Adherence with Dietary and Physical Activity Goals

**Figure 2:**
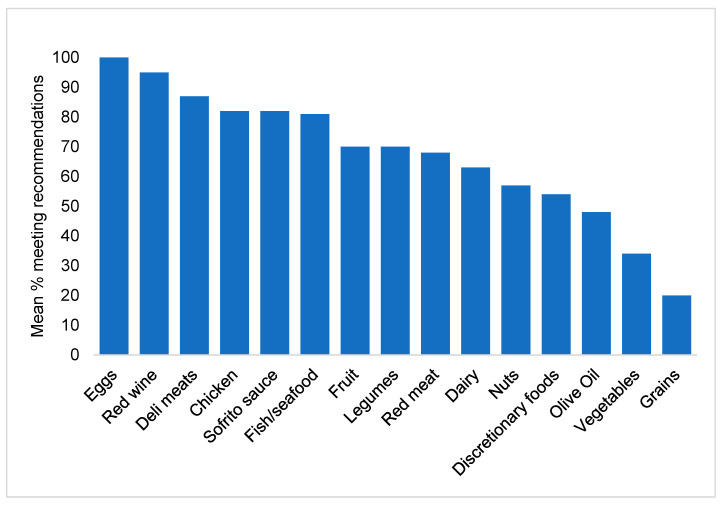
Percentage of participants meeting the dietary recommendations for each food group (mean of all weeks).

Figure 2 shows the percentage of participants who met the dietary recommendation for each food group (the mean across all weeks). Participants often met the recommendations for eggs (100%), red wine (95%), deli meats (87%), chicken (82%), sofrito sauce (82%), and fish/seafood (81%), but struggled to meet the guidelines for olive oil (48%) vegetables (34%), and grains (20%). The mean across all food groups was 67%.

**Figure 3:**
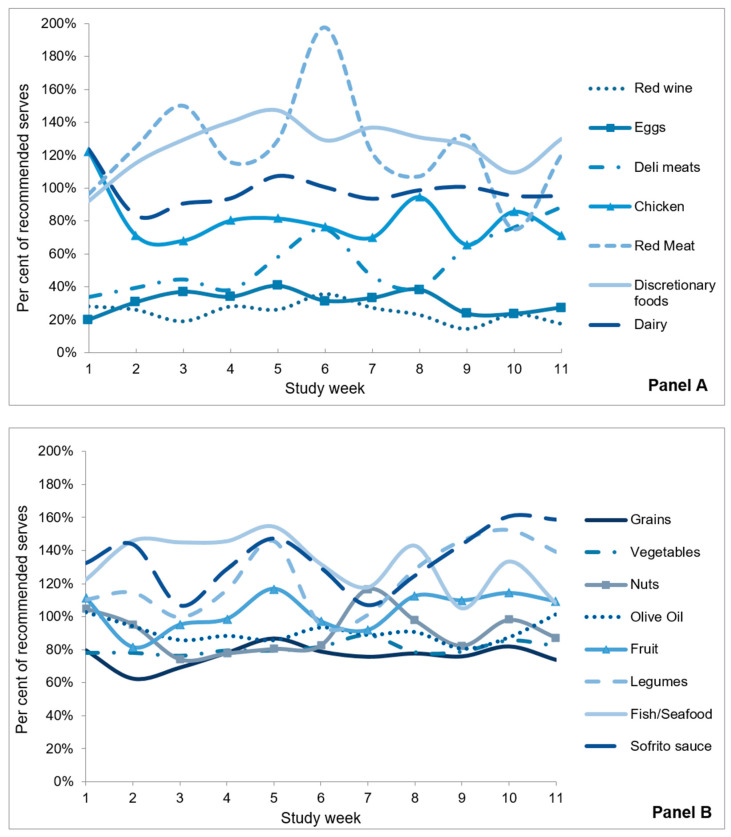
Percentage of recommended dietary servings consumed by study week. Panel (**A**): Foods for which there was a maximum weekly recommendation for serves. Panel (**B**): Foods for which there was a minimum daily or weekly recommendation for serves.

Although participants rarely achieved 100% of the recommendations, average consumption was close to the recommended servings for all food groups. Figure 3 shows the percentage of recommended servings that participants consumed by study week. Participants consumed the minimum recommended number of servings of fruit (104%), nuts (91%), and olive oil (91%), and exceeded the minimum recommended servings of sofrito sauce (135%), fish/seafood (132%), and legumes (123%). Participants consumed fewer than the minimum recommended servings of vegetables (81%) and grain foods (76%).

Participants consumed fewer than the maximum recommended servings of eggs (31%), deli meats (55%), chicken (81%), and red wine (24%), but met the maximum limit for dairy (98%) and exceeded the maximum recommendations for red meat (124%) and discretionary foods (126%).

The average dietary adherence across all food groups was 91%. All foods for which there was a maximum recommendation were reversed scored, and foods for which there was a minimum recommendation were capped at 100% to calculate the average adherence score.

**Figure 4:**
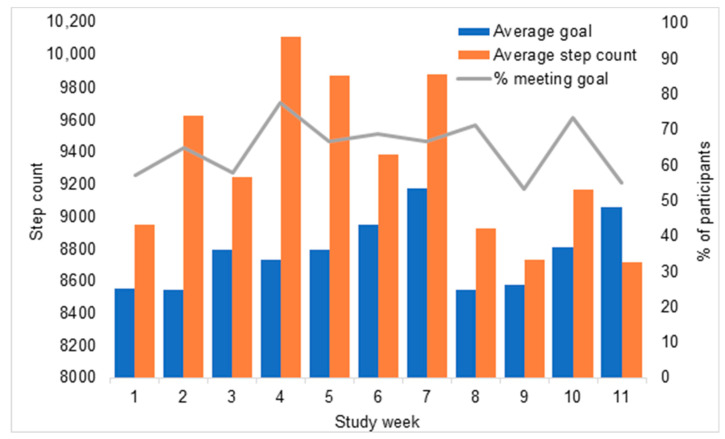
Mean step count, mean step goal, and the percentage of participants who met their goal, by week.

Figure 4 illustrates participants’ mean step count, mean step goal, and the percentage of participants who met their goal, by week. Across all participants, the mean daily step count was 9329 steps. On average, participants exceeded their step goal in all weeks except week 11. However, this was driven by the number of participants who chose steps goals much lower than their average step count. The percentage of participants meeting their step goal was more modest and varied from 53% to 78% across weeks.

### 3.3. Aim 3: User Feedback

The majority of participants completed the user feedback survey at baseline (27 of 31), week 6 (28 of 29), and week 12 (28 of 28).

At baseline, 81% participants agreed they liked the idea of Paola as a virtual health coach. Eighty-nine per cent thought the complexity of the Paola’s language was “about right”, and 100% found the level of formality acceptable. Eighty-one per cent felt comfortable chatting to Paola on Slack.

At week 6, 68% participants reported conversing with Paola only during their scheduled weekly check-ins, while 32% reported conversing with Paola a few times per week. By week 12, 82% participants reported speaking to Paola only during their weekly check-ins, while 17% reported conversing with Paola a few times per week.

At week 6, half of participants provided negative comments about Paola. Complaints related to Paola being frustrating to chat to, not answering questions correctly and not consistently allowing participants to do their weekly check-in when they wanted to. One participant remarked that she was not quite like a human and so it was easier to look things up on Google or on the study website. Another comment was that it was time consuming.

At week 12, 79% of participants agreed that there is potential for chatbots like Paola to help people change their lifestyle, with some qualifications (e.g., “once they get a bit smarter”; “possibly, if its working”; “disguising the automation better would enhance the adoption rate”). Four participants suggested that it might be useful for others, but not for themselves (e.g., “depends on the person”; “others may have found it useful”; “maybe with a younger demographic”; “personally I find talking to chatbots irritating”). Participants were asked how they would improve Paola. Suggestions included giving her more personality, humour, spontaneity such as initiating conversations with participants at random, an increased variety of text response options to questions, enhanced understanding of questions, deeper knowledge, and to provide more personalized feedback during the trial.

### 3.4. Aim 4: Association between Engagement and Program Efficacy

Participants were dichotomized into low and high engagers (≤8 check-ins, *n* = 18 and >8 check-ins *n* = 13, respectively). Based on absolute values, high engagers appeared to make greater improvements in dietary adherence score and MVPA across this program; however, this was not statistically significantly (Table 1).

## 4. Discussion

This 12-week pilot trial of a virtual health assistant-led behavioral intervention resulted in high adherence to the intervention and found no relationship between engagement and study outcomes. The technical performance of the virtual health assistant performed was strong for the weekly check-in sessions with participants, but less successful during spontaneous exchanges. Paola’s intended role was to provide ongoing support outside office hours, in case participants had questions. Interestingly, spontaneous exchanges occurred less often than expected, and in most cases, Paola was unable to respond in a helpful way. When Paola’s question and answer (Q&A) capabilities were developed, we anticipated that participants would want to use this feature of Paola to ask questions related to physical activity and the Mediterranean diet. In reality, few of the spontaneous question’s participants asked Paola were in these realms. Most commonly, participants wanted to ask general dietary advice of Paola, well outside the scope of the study. One participant even asked Paola for a recommendation of where to go shopping in Adelaide to buy an uncommon variety of fish. Participants were disappointed when Paola failed to answer such questions. Whilst Paola’s knowledge base could be extended in future iterations, it is unlikely to be possible to expand her capabilities to answer such specialized questions across such wide-ranging domains. Thus, it may be pertinent to explain Paola’s capabilities during the induction session, to set realistic expectations and avert user disappointment.

Other Q&A failings may be addressed through future enhancements to Paola’s language processing logic. For example, she could be improved by understanding an increased variety of entities, such as the inclusion of more food types, and on more general health topics. Her ability to recognize intents, particularly around “I want”, “is this healthier”, or “I’d like to” should be improved. Participant experience could have been enhanced if she had more responses when she could not understand a question; asking the participant to rephrase the question or to ask it in a different way might have enabled participants to successfully elicit an answer. Improved programming around the intents relating to the check-in and widening the 7-day check-in window would have resolved many errors.

Despite a low spontaneous exchange-rate, by study completion, most participants agreed that a virtual health assistant could be useful in behavior-change programs, suggesting future research in virtual health assistants is warranted. In their mixed-methods evaluation, Nadarzynski et al. [26] interviewed and surveyed adults on their acceptability of chatbots in healthcare. Similarly to this study, the majority of participants were willing to use chatbots for generalized health advice. Further, particpants saw lack of human feeling as a limitation of chatbots, suggesting they were depersonalized and cold, which was reflected in our user feedback. Combined, this feedback implies virtual assistants would be utilized by the general population if they felt “human” enough. In this study we did not find a relationship between engagement and outcomes, which could be explained by the small sample size. Twenty-eight participants completed the study, which may not have provided enough power to detect any difference in diet score or MVPA.

Participants achieved high overall adherence to the program, achieving recommended dietary intakes 67% of the time and meeting their daily step goal nearly 60% of the time. Participants achieved excellent compliance to key Mediterranean diet food groups, such as olive oil, fruit, legumes, fish/seafood, and nuts. These foods contain key nutrients and bioactives, such as flavonoids, fibers, healthy fats like monounsaturated- and long-chain polyunsaturated fatty acids, thought to contribute to the health improvements observed in Mediterranean diet intervention trials [27,28,29,30]. Participants struggled to meet the quantity of bread/cereals recommended, and to limit their discretionary and red meat intakes. These findings highlight areas of focus, such as more support and suggestions to help increase grain intake while decreasing discretionary food intake, which could be programmed into future iterations of Paola.

Overall mean step daily step count was above the mean daily goal; however, this may have been inflated by some participants who exceeded their step goal by a large margin. A more modest 60% of participants met their goal on average. Increased support and instruction for realistic goal setting could improve the program’s effectiveness, as well as improving the number of participants meeting their goal, which could be important for motivation. Although some participants’ goals were too low, mean average steps per day (9600) was well above than the national average for all age groups present in the study (45–54 years, 8093, 55–64 years, 7503, and 65–74 years, 6359 steps per day) [31]. The improvement in step count suggests the program was successful for achieving behavior change and could have long term physical and mental health benefits.

The key strengths of the current study were its novelty. To our knowledge this is the first in-depth process analysis of a natural language processing virtual health coach. We used a mixed-method approach, basing the evaluation on both quantitative server data and qualitative user feedback. Detailed server logs were available, allowing us to scrutinize Paola’s performance in granular detail. This study also had limitations which should be considered. Firstly, the sample size was small, and the exploratory subgroup analyses which we undertook exploring engagement and study outcomes were likely to have been insufficiently powered. If the sample size were larger, analyses could have been split by demographic characteristics, such as sex, age, and socioeconomic status, which may have revealed additional insights into engagement or adherence. Due to a technical error leading to data loss, we were not able to obtain full conversation transcripts for some of the spontaneous exchanges between participants and Paola. This meant we were unable to accurately quantify engagement with this feature of Paola. Furthermore, our analysis on the accuracy of Paola’s responses to spontaneous exchanges is only based on the subset of data we could access. However, it is unlikely that this error would have changed the results substantially. Based on user experience reports, most participants did not interact with Paola outside the weekly check-in for most of the study. There were more females than males in the study, and it appeared that females may have been more willing to engage with the technology as the majority of spontaneous exchanges were from females. Indeed, in a systematic review of 13 studies, 70% of the participants were female, suggesting men are underrepresented in this field of research [17]. Thus, our results cannot be generalized to males. Future research may be needed to address possible gender differences in engagement, which could impact on the design and effectiveness of the virtual health coach.

Future research is needed to address the limitations of Paola, such as knowledge beyond the study scope and inability to respond to requests. Furthermore, this field of research is in its infancy, and learnings from the current study may inform others’ future attempts to design virtual assistants for health behavior change.

Key recommendations for future research are as follows:

- Attention should be paid to the virtual assistant’s “personality”—adding humour and a wider variety of phrasing to answer questions (as a simple example, instead of “yes”, responses such as “absolutely”, “for sure”, and “yes” could be cycled, to produce a more natural, and less robotic conversational style;
- Users want the virtual assistant to initiate regular conversations with them, rather than only weekly. This might be achieved through daily notifications in which the virtual assistant provides tips or asks supportive questions;
- Users appear to have high expectations of virtual assistant’s knowledge and capabilities. Whilst technical advances are constantly improving virtual assistant’s capabilities, some expectation setting during program onboarding may help users set realistic expectations about virtual assistants’ abilities and avoid disappointment;
- Enough resources should be allocated to support intensive, ongoing troubleshooting throughout the trial to allow issues to be addressed in a timely manner, and therefore enhance user experience and trust in the virtual health assistant.

## 5. Conclusions

This study set out to examine the performance, engagement, and acceptability of a physical activity and diet artificial intelligence virtual health assistant. Results showed that the virtual health assistant performed well during the structured weekly check-ins, but displayed performance errors during spontaneous exchanges, due mainly to participants’ queries falling outside Paola’s capabilities. Dietary compliance was high, with participants meeting close to 100% of minimum recommendations for most food groups. Step count goals were met two-thirds of the time. Participants enjoyed the program and felt Paola could be useful in future programs, with improvements. To promote engagement and positive user experience, virtual health assistants need to have a wide knowledge base to meet expectations of users, a sophisticated ability to recognize intents and entities, personality, and spontaneity. Ongoing technical troubleshooting of the virtual assistant and setting realistic user expectations during onboarding is also recommended.

## Figures and Tables

**Table 1:** Descriptive statistics and results from linear mixed models examining the association between engagement with Paola and change in dietary adherence score and moderate-to-vigorous physical activity (MVPA) from baseline to week 12.

	Low Engagers (Mean ± SD)		High Engagers (Mean ± SD)		Time × Engagement Interaction	
	Baseline (*n* = 18)	Week 12 (*n* = 15)	Baseline (*n* = 13)	Week 12 (*n* = 13)	B [95% CI]	*p*
Dietary score	3.8 ± 1.7	9.7 ± 1.4	3.8 ± 2.0	10.8 ± 3.1	1.2 [−0.8–3.2]	0.255
MVPA	159 ± 115	258 ± 240	272 ± 206	418 ± 227	48.6 [−126.5–223.7]	0.587

B = Beta coefficient, 95% CI = confidence interval.

## Supplementary Materials

The following are available online at https://www.mdpi.com/1660-4601/17/23/9137/s1, Table S1: Spontaneous exchanges related to diet; Table S2: Spontaneous exchanges related to physical activity; Table S3: Spontaneous exchanges related to the program; Table S4: Spontaneous exchanges related to other themes.

## References

[1] World Health Organisation Diet, Nutrition and the Prevention of Chronic Diseases: Report of the Joint WHO/FAO Expert Consultation. https://www.who.int/dietphysicalactivity/publications/trs916/summary/en/.

[2] Australian Institute of Health and Welfare, Australian Government (2015). Australian Burden of Disease Study: Impact and Causes of Illness and Death in Australian 2015—Summary Report.

[3] Australian Health Survey: Nutrition First Results—Foods and Nutrients. https://www.abs.gov.au/statistics/health/health-conditions-and-risks/australian-health-survey-nutrition-first-results-foods-and-nutrients/latest-release.

[4] National Health Survey: First Results, 2017–2018. https://www.abs.gov.au/statistics/health/health-conditions-and-risks/national-health-survey-first-results/latest-release.

[5] Australian Institute of Health and Welfare, Australian Government, Australian Institute of Health and Welfare (2018). Australia’s Health 2018.

[6] Stein N., Brooks K. (2017). A fully automated conversational artificial intelligence for weight loss: Longitudinal observational study among overweight and obese adults. JMIR Diabetes.

[7] Laranjo L., Dunn A.G., Tong H.L., Kocaballi A.B., Chen J., Bashir R., Surian D., Gallego B., Magradbi F., Lau A.Y.S. (2018). Conversational agents in healthcare: A systematic review. JAMIA Open.

[8] Stephens T.N., Joerin A., Rauws M. (2019). Feasibility of pediatric obesity and prediabetes treatment support through Tess, the AI behavioral coaching chatbot. TBM.

[9] Hauser-Ulrich S., Kunzli H., Meier-Peterhans D., Kowatsch T. (2020). A smartphone-based health care chatbot to promote self-management of chronic pain (SELMA): Pilot randomised controlled trial. JMIR mHealth uHealth.

[10] Maeda E., Miyata A., Boivin J., Nomura K., Kumazawa Y., Shirasawa H., Saito H., Terada Y. (2020). Promoting fertility awareness and preconception health using a chatbot: A randomised controlled trial. RBMO.

[11] Gabrielli S., Rizzi S., Carbone S., Donisi V. (2020). A chatbot-based coaching intervention for adolescents to promote life skills; Pilot study. JMIR Hum. Factors.

[12] Piao M., Ryu H., Lee H., Kim J. (2020). Use of the healtgy lifestyle coaching chatbot app to promote stair-climbing habits among office workers: Exploratory randomized controlled trial. JMIR mHealth uHealth.

[13] Fitzpatrick K.K., Darcy A., Vierhile M. (2017). Delivering cognitive behaviour therapy to young adults with symptoms of depression and anxiety, using a fully automated conversational aged (Woebot): A Randomized Controlled Trial. JMIR Ment. Health.

[14] Bickmore T.W., Schulman D., Sidner C. (2013). Automated Interventions for Multiple Health Behaviours Using Conversational Agents. Patient Educ. Couns..

[15] Bickmore T.W., Silliman R.A., Nelson K., Cheng D.M., Winter M., Henault L., Paasche-Orlow M.K. (2013). A randomized controlled trial of an automated exercise coach for older adults. J. Am. Geriatr. Soc..

[16] Kocielnik R., Xiao L., Avrahami D., Hsieh G. (2018). Reflection Companion: A conversational system for engaging users in reflection on physical activity. Proc. ACM Interact. Mob. Wearable Ubiquitous Technol..

[17] Gaffney H., Mansell W., Tai S. (2019). Conversational agents in the treatment of mental health problems: Mixed method systematic review. JMIR mHealth uHealth.

[18] Zhang J., Oh Y.J., Lange P., Yu Z., Fukuoka Y. (2020). Artificial intelligence chatbot behaviour change model for designed artificial intelligence chatbots to promote physical activity and a healthy diet: Viewpoint. J. Med. Internet Res..

[19] Maher C.A., Davis C.R., Curtis R.G., Murphy K.J. (2020). A Physical Activity and Diet Program Delivered by Artificially Intelligent Virtual Health Coach: Proof-of-Concept Study. JMIR mHealth uHealth.

[20] Davis C.R., Hodgson J.M., Bryan J., Garg M., Woodman R., Murphy K. (2017). Older Australians can achieve high adherence to the Mediterranean diet during a 6-month randomised intervention; results from the MedLey study. Nutrients.

[21] Australian Institute of Health and Welfare (AIHW) (2003). The Active Australia Survey: A Guide and Manual for Implementation, Analysis and Reporting.

[22] Brown W., Burton N., Marshall A., Miller Y. (2008). Reliability and validity of a modified self-administered version of the Active Australia physical activity survey in a sample of mid-age women. Aust. N. Z. J. Public Health.

[23] Schröder H., Fitó M., Estruch R., Martínez-González M., Corella D., Salas-Salvadó J. (2011). A short screener is valid for assessing Mediterranean diet adherence among older Spanish men and women. J. Nutr..

[24] Wade A., Davis C., Dyer K., Hodgson J., Woodman R., Murphy K. (2018). A Mediterranean diet supplemented with dairy foods improves markers of cardiovascular risk: Results from the MedDairy randomized controlled trial. Am. J. Clin. Nutr..

[25] Guide to Mobile Notification. https://slack.com/intl/en-au/help/articles/360025446073-Guide-to-mobile-notifications.

[26] Nadarzynski T., Miles O., Cowie A., Ridge D. (2019). Acceptability of artificial intelligence (AI)-led chatbod services in healthcare: A mixed-methods study. Digit. Health.

[27] Davis C.R., Hodgson J.M., Woodman R., Bryan J., Wilson C., Murphy K.J. (2017). A Mediterranean diet lowers blood pressure and improves endothelial function: Results from the MedLey randomized intervention trial. Am. J. Clin. Nutr..

[28] Serra-Majem L., Roman B., Estruch R. (2006). Scientific evidence of interventions using the Mediterranean diet: A systematic review. Nutr. Rev..

[29] Estruch R., Ros E., Salas-Salvadó J., Covas M., Corella D., Arós F., Gomez-Gracia E., Ruiz-Gutierrez V., Fiol M., Lapetra J. (2018). Primary Prevention of Cardiovascular Disease with a Mediterranean Diet Supplemented with Extra-Virgin Olive Oil or Nuts. N. Engl. J. Med..

[30] De Lorgeril M., Renaud S., Salen P., Monjaud I., Mamelle N., Martin J., Guidollet J., Touboul P., Delaye J. (1994). Mediterranean alpha-linolenic acid rich diet in secondary prevention of coronary heart disease. Lancet.

[31] Australia Bureau of Statistics (ABS) (2013). Australian Health Survey: Physical Activity, 2011–2012.

