# Supplementary material for: A Process Evaluation Examining the Performance, Adherence, and Acceptability of a Physical Activity and Diet Artificial Intelligence Virtual Health Assistant
Source: Int J Environ Res Public Health. 2020 Dec 7;17(23):9137. doi: 10.3390/ijerph17239137 (PMC7729471; doi:10.3390/ijerph17239137)
Supplement: Supplementary file 1 [file ijerph-17-09137-s001.pdf]

Table S1. Spontaneous exchanges related to diet

Participant	Paola's response	Appropriate (Y/N)
1. How can I increase my intake of olive oil?	Try to use more in cooking, more than you would usually use. Use it in baking sweet foods as well, and use olive oil for your salad dressings, dips and on bread. Don't be scared to use it abundantly!	Y
2. Are baked beans considered lentils?	Legumes include several bean varieties, chickpeas and lentils of all types. Cannellini beans, kidney beans, black beans, broad beans, baked beans and lima beans are all considered legumes. You should aim to have approximately 250 grams of legumes per week.	Y
3. Green vegetables?	You can have all types of vegetables. Try to include a variety and avoid juice if possible. It is important to include tomatoes, onions, garlic and leafy green vegetables (salads) in your diet. You can have any others as well, such as capsicum, zucchini, eggplant, cucumber, cruciferous vegetables such as broccoli and cauliflower, and potato.	Y
4. Pumpkin?	You can have all types of vegetables. Try to include a variety and avoid juice if possible. It is important to include tomatoes, onions, garlic and leafy green vegetables (salads) in your diet. You can have any others as well, such as capsicum, zucchini, eggplant, cucumber, cruciferous vegetables such as broccoli and cauliflower, and potato.	Y
5. Is curry healthy?	Are there any other questions you have about the Mediterranean diet or any concerns or challenges you've had?	N
6. Are curries healthy?	Are there any other questions you have about the Mediterranean diet or any concerns or challenges you've had?	N
7. Is miso healthy?	-	N
8. Is miso good for you?	Sorry, I'm not sure how to answer that. If you still have concerns, please have a look at <website> or contact our team.	N
9. Is Miso paste good for you?	-	N
10. Where do liver and kidneys belong?	Are there any other questions you have about the Mediterranean diet or any concerns or challenges you've had?	N
11. What about liver and kidneys?	Are there any other questions you have about the Mediterranean diet or any concerns or challenges you've had?	N
12. What suggestions do you have re legumes?	Sure!	N
13. Ideas with using lentils?	Are there any other questions you have about the Mediterranean diet or any concerns or challenges you've had?	N
14. Ideas for using lentils?	Are there any other questions you have about the Mediterranean diet or any concerns or challenges you've had?	N

15. Is coconut milk considered fruit?	All types of fruit are fine to consume on the Mediterranean diet, fresh, canned or dried. Try to have 2 or 3 serves of fruit every day.	N
16. Any tips on where I can source coral trout in Adelaide, I am not having much luck?	You should have oily or fatty fish at least once per week, and in total, you should have fish or seafood 3 times per week. Try salmon, mackeral, trout or sardines for your oily fish serve, and canned tuna, or prawns, for your other serves	N
17. How much water should you drink each day?	Are there any other questions you have about the Mediterranean diet or any concerns or challenges you've had?	N
18. I don’t like eating 1 cup of rice or pasta, what else can I try?	Are there any other questions you have about the Mediterranean diet or any concerns or challenges you've had?	N
19. Yes. I have eaten too many discretionary foods this week	Sure, what can I help you with?	N
20. information about grain types and portions was useful	For a full list of foods and food groups, visit the following page on the MedLiPal website <website>	N

Table S2. Spontaneous exchanges related to physical activity

**Participant**	**Paola’s response**	**Appropriate (Y/N)**
21. How can I increase my step count?	There are many ways you can add steps to your day! Many activities and exercises add steps, such as walking, jogging or playing golf. Think of ways you can make small increases throughout your day, for example by taking an extra walk, walking for transport, taking the 'long way' to get somewhere, doing chores around the house, using stairs, or getting out into the garden a bit more. Don't be discouraged, it takes time to build up habits and change your behaviour! If you do lots of activities but don't do many steps (for example, you cycle or swim a lot), it is ok to have a lower step goal! Just set a good step goal to reflect your lifestyle, and keep up the good work being active.	Y

Table S3. Spontaneous exchanges related to the program

Participant	Paola's response	Appropriate (Y/N)
22. Weekly check-in	Great idea, let me see if your next checkin is due ...	Y
23. May I checkin Paola	Great idea, let me see if your next checkin is due ...	Y
24. Can I please do my weekly checkin	Great idea, let me see if your next checkin is due ...	Y
25. Ready to do weekly check-in	Great idea, let me see if your next checkin is due ...	Y
26. start checkin	Hi , I'm Paola. Are you ready to complete your weekly check-in? This will take around 15 minutes. You can also ask me any questions you have about the diet/activity at this time!	Y
27. weekly check-in start	Are you ready to complete your weekly check-in? This will take around 15 minutes. You can also ask me any questions you have about the diet/activity at this time!	Y
28. Redo checkin	I have reset your weekly check-in so that you can check-in again.	Y
29. Check-in	You need to check in weekly. Therefore you should have 11 check-ins throughout the study. Your first check-in should be at the end of your first week.	N
30. Do check in	You need to check in weekly. Therefore you should have 11 check-ins throughout the study. Your first check-in should be at the end of your first week.	N
31. I would like to check in	You need to check in weekly. Therefore you should have 11 check-ins throughout the study. Your first check-in should be at the end of your first week.	N
32. I want to check-in	You need to check in weekly. Therefore you should have 11 check-ins throughout the study. Your first check-in should be at the end of your first week.	N
33. please can I check in now	You need to check in weekly. Therefore you should have 11 check-ins throughout the study. Your first check-in should be at the end of your first week.	N
34. weekly sign in	-	N
35. Check in did not allow me to respond and input answers from previous questions automatically	You need to check in weekly. Therefore you should have 11 check-ins throughout the study. Your first check-in should be at the end of your first week.	N

36. How do I change this weeks checkin information?	You need to check in weekly. Therefore you should have 11 check-ins throughout the study. Your first check-in should be at the end of your first week.	N
37. Only a minor frustration at accessing the check in process	You need to check in weekly. Therefore you should have 11 check-ins throughout the study. Your first check-in should be at the end of your first week.	N
38. I have made a mistake how do we start again?	Next I'll ask you in WEEK totals. During the past WEEK, how many SERVES of nuts have you had? (1 handful = 1 serve)	N
39. In the survey you did not ask me to input average servings for vegetables	Yes, sure thing.	N
40. oops sorry Paola I gave you average not total for the week of nuts. I had 14 SERVES of nuts this week.	Well done! You have had 14 serves of legumes this week. You are on track following the Mediterranean diet.	N
41. Survey didn't run well	Are there any other questions you have about the Mediterranean diet or any concerns or challenges you've had?	N
42. I've printed them off.	-	N

Table S4. Spontaneous exchanges related to other themes

**Participant**	**Paola's response**	**Appropriate (Y/N)**
43. I had an infection & tooth removed so needed to rest	Sorry, I am unable to answer that at this time... I have taken a note of it for next time. If you still have concerns, please have a look at <website> or contact our team.	Y
44. The weather has been cold and wet	Sorry, I am unable to answer that at this time... I have taken a note of it for next time. If you still have concerns, please have a look at <website> or contact our team.	Y
45. I was sick 3 days this week which is why I didn't reach my target	Sorry, I am unable to answer that at this time... I have taken a note of it for next time. If you still have concerns, please have a look at <website> or contact our team.	N
